# Dr. Bany, on Vaccine Inoculation

**Published:** 1800-06

**Authors:** John Bany

**Affiliations:** Cork


### *To Dr.* BRADLEY.

Sir,

As an old fellow-ftudent, I take the liberty of applying to to you to fend me fome of the *matter* of cow-pox, which I find by your ufeful Journal, has been conveyed to different parts in letters. I have made many inquiries in this neighbourhood to procure it; but as the complaint has not appeared lately among the dairies, I have not been fortunate enough to meet it. I find, however, that the country people are well acquainted with the difeafe, and defcribed it very accurately to me. It is known by the Irifh name of (Shinagh). They have long attributed to it the anti-variolous power, which renders it fo important a difcovery to the happinefs of mankind. I met with two people, who had been themfelves affected with the complaint. A lady fhewed me the mark of it in one of her hands. She had it about forty years fince, and was then informed by fome of her neighbours that fhe never would have the fmall-pox; but gave little credit to their affertions. She has, however, been fince frequently expofed to the infection, and in confequence of her incredulity received feveral frights, particularly during the illnefs of her children, who had the fmall-pox rather heavily; but though fhe attended them very clofely through the whole of their complaint, fhe did not take it herfelf. The other perfon is a gardener, who lives with a country gentleman of my acquaintance. He gave himfelf the cow-pox purpofely by rubbing himfelf againft fome perfon who was affected with it, from a conviction that it would prevent the fmall-pox. This happened feveral years ago; and though he has often put himfelf in the way of the fmall-pox infection, and even lain in the fame bed with his children when they were covered with it, he has not taken the difeafe. If I had time to make the neceffary inquiries, I am fure I could multiply inftances of this kind, as I heard of many others who had the cow-pox, and efcaped the fmall-pox in the fame manner. But as I faw and converfed with thefe two, who are people of undoubted veracity, I believe they will prove sufficiently satisfactory to you. May I expect the pleasure of a letter from you shortly with the *vaccine matter* inclosed? Your Journal is taken in by many of the faculty in this city. I hope in the course of time to be able to transmit to you some useful facts for insertion in it. I am,

Sir,

Your obedient humble servant,

*Cork, April 20, 1800.* JOHN BANY.

